# Novel Poly(ionic liquid) Augmented Membranes for Unconventional Aqueous Phase Applications in Fractionation of Dyes and Sugar

**DOI:** 10.3390/polym13142366

**Published:** 2021-07-19

**Authors:** Sandrina DePaz, Arijit Sengupta, Yu-Hsuan Chiao, Sumith Ranil Wickramasinghe

**Affiliations:** 1 Ralph E Martin College of Chemical Engineering, University of Arkansas, Fayetteville, AR 72701, USA; ssdepaz@email.uark.edu (S.D.); msdonaldc@hotmail.com (Y.-H.C.); swickram@uark.edu (S.R.W.); 2 Radiochemistry Division, Bhabha Atomic Research Centre, Mumbai 400085, India; 3 HomiBhabha National Institute, Mumbai 400094, India

**Keywords:** poly(ionic liquids), membrane, mass transfer, sugar fractionation, dyes

## Abstract

Poly(ionic) liquid (PIL) augmented membranes were fabricated through self-polymerization of 2-vinyl pyridine and 4-vinyl pyridine followed by dopamine triggered polymerization and bridging with inert polyamide support. The resulting membranes acquired a positive surface charge with a high degree of hydrophilicity. Fourier transformed Infra-red (FTIR) and Energy dispersive X-ray (EDX) spectroscopic investigation revealed the successful augmentation of PIL surface layer, whereas surface morphology was investigated through scanning electron microscopy (SEM) imaging. This manuscript demonstrates pi electron-induced separation of dyes with the trend in permeability: Coomassie Brilliant Blue G (CBBHG) > Remazol Brilliant Blue R (RBBR) > Eichrome Black T (EBT) > Congo Red (CR). CBBG exhibited extended conjugation over large aromatic domain. RBBR and EBT were associated withtheelectron-donating -NH_2_ group and electron-withdrawing -NO_2_ group, respectively, hence pi electron density on aromatic ring varied. The steric repulsion between two pairs of ortho hydrogens (Hs) in biphenyl moieties of CR resulted in deviation of planarity and hence aromaticity leading to the lowest permeability. The sugar fractionation followed the trend: Galactose > Mannose > Fructose > Glucose > Xylose. More hydroxyl (-OH) groups in sugars and their conformational alignment in the same direction, exhibited more lone pair of electrons leading to more interaction with PIL and hence better permeability. Pentose showed poorer permeation than hexose, whereas aldose showed better permeation than ketose.

## 1. Introduction

Ionic liquids (ILs) have been a subject of interest in recent decades due to their very low volatility, non-flammability, high heat capacity, solvating power, polarizing ability, high ionic conductivity, chemical, radiolytic and thermal stability [1,2,3,4]. ILs are organic salts, which gained a reputation as an environmentally friendly or novel “green” solvent to potentially replace volatile organic diluents [4,5,6]. Moreover, the uniqueness of ionic liquids prompt advancement in the fields of synthesis, catalysis, separations, electrochemistry, energy, nuclear science, hydrometallurgy, biotechnology, water treatment and biomass processing [7,8,9,10,11]. Their customizability of chemical and physical properties through different combinations of cations and anions are also advantageous [11]. Small adjustments to the composition of ILs can cause a great change in physiochemical properties and in turn provides room for refinement [12,13]. Common IL cations include imidazolium, pyridinium, alkylammonium and alkylphosphonium; to be paired with inorganic anions such as halides, mineral acids, polyatomic inorganic, etc. [14]. A major economic drawback for the use of ionic liquids istheir high synthesis cost [15]. Another challenge is developing equally “green” processes for solute recovery as large amounts of energy are needed [16]. However, the implementation of ionic liquid membranes should reduce cost drawbacks. As per recovery, extraction with carbon dioxide and pervaporation have shown promising results [17]. The supported ionic liquid membranes (SILMs) are unstable due to vaporization, dissolution and/or shifting of the solvent embedded in the supporting membrane [18]. The issue of long-term performance and stability of SLMs has affected industrial applications [19]. ILMs are favorable in a variety of applications for their tunable properties, high selectivity and negligible loss of ionic liquid [19]. SILMs were reported to effectively separate organic compounds based on π electron density [20,21,22]. The SILM stability is primarily affected by preparatory methods, properties of the support material, and IL to support membrane compatibility. Ionic liquid loss is due mainly to pore extrusion and compression [23]. Furthermore, the critical concern of IL water solubility still remains and their toxicity toward a range of organisms and enzymes can limit their use in extractions during fermentation [24,25].

Polymers of ionic liquids, which contain repetitive polymer chain units, are known as poly(ionic liquid)s (PILs) [26]. These polymeric absorbents have shown to be promising alternatives to organic liquids and ionic liquids, owing to their viscoelastic solid properties and ability to be tuned for water insolubility [27]. PILs are synthesized through polymerization of IL monomers or chemical modification of polymeric precursors. Their properties can be controlled provided the simplicity of ion-exchange reactions [28]. Uniquely, PIL should combined property effects of their monomeric counterpart. Primary applications of PILs thus far include gas capture, sorption, wastewater treatment, catalysis, hydrogen energetic, electrochemistry, bio- and membrane technology [29,30,31,32]. Further research and implementation of polyionic liquids as novel antibacterial agents can decrease antibiotic drug resistance [33,34]. Grafting PIL brushes on the membrane surface was reported to exhibit the electrical response in presence of external oscillating electric field [35,36]. A pressure-assisted self-assembly method was used to deposit the selective poly(ionic) liquid layer onto the polyimide support [37]. Concerns regarding the stability of the PIL membrane in aqueous and polar media arose after tests revealed that the conductivity increased rapidly in the presence of water, demonstrating the detachment of the PIL surface coat [37]. In the present research, polyimide support membranes have been augmented with poly(ionic liquid) using a dopamine triggered polymerization and co-deposition method to provide a selective surface layer [38]. Dopamine or polydopamine chemistry has introduced the fabrication of high-performance membranes with improved fouling resistance and increased hydrophilicity [39,40,41]. The basis of separation proposed is the pi electron cloud affinity between the PIL and chemical species in the feed. This paper will focus on the PIL membrane applications in sugar and dye fractionation/separation.

Membrane technology for sugar fractionation is important in the food industry and in the recovery of saccharides from fermentation, hydrolysis, chemical and biofuel production [42]. However, difficulty to fractionate mono- and di- saccharides with a separation factor greater than 3 has been faced [43]. Increasing sugar concentration in bioreactors is desired during fermentation processes for a greater product yield [43,44]. New economic processes are required for lignocellulosic biomass hydrolysis during the production of 2nd generation bio-fuels and chemical intermediates [45,46,47,48]. All sugars tested in present investigation were monosaccharides including glucose, fructose, mannose, galactose and xylose with the exception of sucrose being an oligosaccharide. Dye removal is of great interest to the textile industry, being one of the few large industries that consume significant volumes of water [49,50,51,52,53,54]. Membrane separation is employed as the tertiary stage in the wastewater treatment. Commercial dying processes mainly use reactive dyes and some amounts of water soluble and insoluble organic dyes [55]. Common synthetic dyes which are utilized in current research include Congo Red, Eichrome Black T, Coomassie Brilliant Blue G and Remazol Blue R. These dyes are low in cost and high in color longevity [56]. The possession of complex molecular structures by the synthetic dyes urges the necessity for inexpensive water treatment solutions [56].

## 2. Materials and Methods

### 2.1. Materials

The following reagents were of analytical grade purity, unless noted otherwise. The2-vinyl imidazole, 4-vinyl imidazole, allyl bromide, Dopamine Hydrochloride, Congo Red (CR), Coomassie Brilliant Blue G (CBBG), Remazol Brilliant Blue R (RBBR), Eichrome Black T (EBT), D(+)-Glucose, D(+)-Xylose, D(+)-Mannose, D(+)-Galactose, D(+)-Sucrose and D(−)-Fructose were acquired from Sigma Aldrich (Munich, Germany). A summary of physiochemical properties is included in Table 1. Tris(hydroxymethyl)aminomethane (Tris)-HCl was procured from Amresco Inc. (Solon, OH, USA). Polyimide ((P84) was provided by HP Polymer GmbH (Lenzing, Austria). Remaining reagents such as Dimethylsulfoxide (DMSO), 1-6-Hexamethylenediamine (HMD), Polyethylene Glycol 400 (PEG400), Isopropyl Alcohol, Hydrochloric acid (HCL) and Ethanol were obtained from Seastar Chemicals Inc. (Sidney, DC, Canada).

### 2.2. Instrumentations

Deionized water used in experiments was produced with a Thermo Fisher 18 MΩ Barnstead Smart2Pure system (Schwerte, Germany). Membrane casting was performed with a 200 µm non-woven knife provided by the Membrane Science Inc. (Hsinchu, Taiwan). The membranes were dried using a414004-580 VWR Symphony Vacuum Oven procured from VWR (Radnor, PA, USA). Water contact angle measurements utilized a sessile drop contact angle goniometer (Model 100) purchased from Rame-Hart Instrument Company (Netcong, NJ, USA). Dead-end nanofiltration experiments were conducted using a HP4750 Sterlitech Stirred Cell procured from SterlitechCooperation (Kent, WA). Permeate flux data wererecorded at 30 s intervals using the Balinx Flux Program and a JL1502 Mettler Toledo balance (Columbus, OH, USA). Conductivity was measured using a conductivity meter procured from VWR, (Radnor, PA, USA). Scanning electron microscopy (SEM) Images were taken using a Nova Nanolab 200 Duo-Beam Workstation procured from FEI Company (Hillsboro, OR, USA). The same instrument was used for Energy Dispersive X-ray (EDX) spectroscopic analysis. Fourier transform infrared (FTIR) spectroscopic data wereobtained from IR Affinity with a wavenumber range of 7800–350cm^−1^ and a horizontal ATR ZnSe accessory, acquired from Shimadzu (Columbia, MD, USA). UV-Vis analysis of the dye compounds was assessed using the GENESYS 10S UV-Vis Spectrophotometer obtained from Thermo Fisher Scientific (Waltham, MA, USA).

### 2.3. Fabrication of the PIL Augmented Membranes

#### 2.3.1. Synthesis of Poly(ionic) Liquid

One pot synthesis of the PILs were carried out as described in the previous literature [34]. A mixture containing 2-vinyl pyridine (2.5 mol) and allyl bromide (2.5 mol) was stirred on a temperature-controlled shaker at 333K. A stirring speed of 100rpm was maintained for30 min. Separation resulted in a yellow polymeric solid. The solid was then washed three times with ethyl acetate for the removal of any unreacted precursors. The mixture of 4-vinyl pyridine (50mmol) and allyl bromide (50 mmol) was prepared in a similar method but stirred for 2.5 h at 100rpm. The polymeric solid obtained from 4-vinyl pyridine was light pink in comparison. Both solids were dried and scraped with a spatula until a powdered texture was obtained. Finally, the powders were stored in a sealed glass container till use for preparation of a liquid solution.

#### 2.3.2. PIL augmentation

Synthesis of Polyimide Support: A 24% (*w/w*) solution of polyimide (P84) was stirred and degassed in DMSO for 24 h. The solution was then cast on polyester using a non-woven casting knife. Post casting, the membrane was placed in a water bath to undergo phase inversion. *Iso*-propanol was used to wash the membrane and remove residual water. Cross-linking was carried out using HMD in isopropyl alcohol for 16 h at room temperature. The membrane was then immersed in *iso*-propanol for 3 h to removeexcess cross-linking agent. Thereafter, the membrane was immediately submerged in a 3:2 ratio of polyethylene glycol 400 to *iso*-propanol [57]. Lastly, the resulting polyimide support was wiped with tissue paper and left to dry overnight.

*Fabrication of PIL augmented membranes:* The fabrication of PIL augmented membranes was carried out in two steps. The first step involved the preparation of porous inert polyimide support. The second step was to synthesize and incorporate the PIL through covalently bonded dopamine. The polyimide support membranes were dried with tissue paper and each separately placed, with the active side facing upward, into a 250 mL conical flask along with the deposition solution. The flask was set on an orbital shaker for 20 h at 24 °C [34]. During shaking, a color change from orange to brown then black was observed. Thereafter, the membrane was gently removed from the flask. The membrane was rinsed thrice with DI water and thrice with 50% ethanol water. Finally, the PIL modified membrane was stored in DI water until further use. This method was repeated for each membrane modification using 4-vinyl pyridine or 2-vinyl pyridine PIL [37]. Over time the conductivity of the DI water, in which the membranes were stored, was measured using a conductivity meter from VWR and no significant increase was shown.

### 2.4. Characterization

#### 2.4.1. Water Contact Angle

Prior to contact angle measurement, the membranes were dried in a VWR symphony vacuum oven, procured from VWR, at 60 °C for 3 h. The drop shape analysis system was utilized for contact angle measurement of the membrane. First, a micropipette was filled with water and clamped to the device. Adjustments to the focus and illumination were performed using the analysis software. A drop of water (20 μL) was deposited onto the membrane at room temperature and the contact angle was measured by the camera. The software automatically analyzed the water drop as a function of time.

#### 2.4.2. FTIR

Surface modification of the membranes were assessed using FTIR spectra to identify key functional groups. Using the Shimadzu IR spectrophotometer, data wereobtained from approximately 884 scans which covered a range of 598 to 4000 cm^−1^. Spectra of the base membrane, 2-vinyl PIL and 4-vinyl PIL were compared.

#### 2.4.3. SEM

Scanning electron microscopy images of the modified and unmodified membranes were captured to visualize the pores filled with poly(ionic) liquid. The samples were immersed in ethanol/water mixtures to remove contaminants, rinsed with DI water, then dried prior to analysis. Membrane samples were further prepared by cutting out small squares. The samples were then attached to a zinc-selenium plate and sputtered with gold molecules.

#### 2.4.4. EDX

Energy Dispersive X-Ray Spectroscopy (EDX) was used to examine the chemical composition and morphological appearance. EDX facilitates identification of elements and their percent distribution by weight. PIL membrane samples were subjected to a focused beam of electrons and therein emit an X-ray spectrum for restricted qualitative chemical analysis.

### 2.5. Performance

The filtration experiments were carried out following our earlier reported procedure [37]. Similar experimental parameters were used for the filtration as given below.

#### 2.5.1. Filtrationof Dyes

The membrane performance was tested by series of dye filtration experiments conducted at 50 psi and 24 °C. All dead-end filtrations were carried out in a large pressurized HP4750 Sterlitech Stirred Cellfiltration system. A 30mL solution with equal concentrations of Brilliant Blue G, Remazol Brilliant Blue R (RBBR), Coomassie Brilliant Blue G (CBBG) and Eichrome Black T (EBT) was loaded into the cell along with the augmented membrane. The membrane used in the present case was in the form of a circular disc having 4.7 cm diameter with effective surface area 17.35 cm^2^. The glass container was used to collect the filtrate. For each, 2mL of three permeate solutions were collected for determination of dyes by UV-VIS spectrometry. Dye filtrations were performed using the 2-PIL and 4-PIL membrane separately.

#### 2.5.2. Filtration of Sugars

Similar methods were conducted for the filtration of sugars. HPLC method has been used for the determination of sugar. Organic Acid Analysis Aminex HPX-87H ion exclusion column (300 × 7.8 mm), procured from BioRad, Hercules, California, USA was used for sugar analyses. The temperature of the column was maintained at 65 ± 0.1 °C. The mobile phase used for the present case was maintained at pH 2.28 using sulfuric acid, while the flow rate was kept 0.65 mL/min. The solvent delivery system was a Waters 515 HPLC pump (procured from Milford, Massachusetts, United States) equipped with a Waters 717 plus auto sampler. Refractive index of the sugars was measured using a Waters 410 differential refractometer. Sugar content was determined from the linear regression of the standard curves of glucose, fructose, and mannose which ran at 1.25, 0.625, 0.313, 0.156, 0.078, 0.039 g/100mL.

## 3. Results

### 3.1. PIL Augmentation on Polyimide Membrane and Their Characterization

#### 3.1.1. Synthesis of Poly(ionic) Liquid 

**Figure 1:**
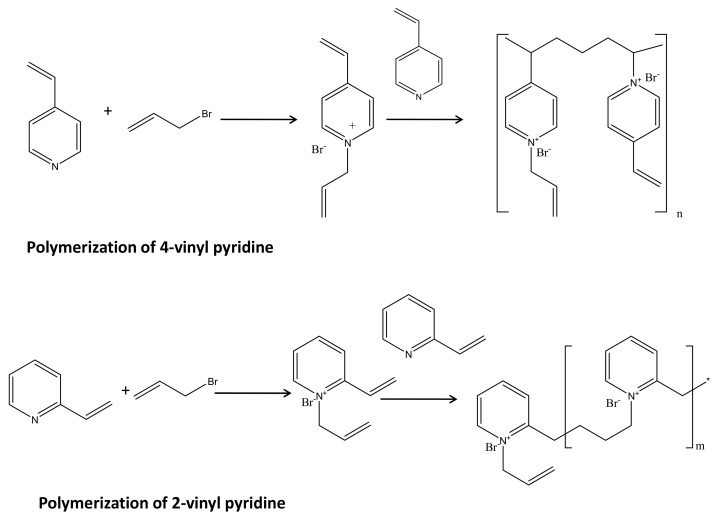
The schematic presentation of preparation of poly(ionic liquid).

In this step, two chemical reactions occurred simultaneously in one pot. The allyl bromide has a tendency to release bromide ion and forming allylic cation [34]. The allylic cation is having two same canonical structure and hence highly resonance stabilized. The lone pair of electrons on pyridine moiety is not a part of aromatic system (unlike pyrrole), therefore relatively free for coordination. The querternization occurs by pyridine N to allylic moieties resulting N-allyl, 2 vinyl pyridinium bromide (Menshutkin reaction) [37]. Based on our earlier literature, this compound in presence of 2 vinyl pyridine undergoes polymerization resulting poly(ionic liquid) polymers. The molecular weight of such polymers has been reported earlier. The similar polymerization happened for 4-vinyl pyridine also. The polymerization predominantly might proceed through bimolecular or uni-molecular or a combination of both. The mechanism is not still unambiguous. There might be a chance of self-polymerization of vinyl pyridine, however, our earlier study demonstrated that due to the more electron density on allylic moieties in vinyl pyridinium bromide, the allylic group actively took part in the polymerization [34,37]. Figure 1 schematically presented the first step of the membrane fabrication. Poly(2 vinyl pyridinium) bromide poly(ionic liquid) was reported to be mono-dispersed polymer with 20 kDa molecular weight, whereas 4 vinyl pyridinium bromide poly ionic liquid was reported to form 60 kDa polymer in more than 95% yield, while less than 5% poly(ionic liquid) was reported to have less than 6 kDa molecular weight [34]. 

#### 3.1.2. Fabrication of Active Surface Layer

**Figure 2:**
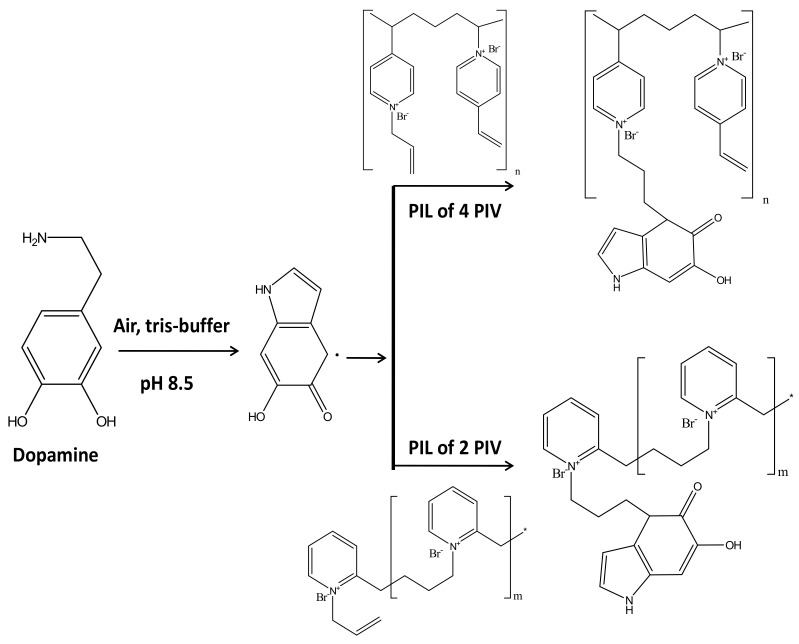
The schematic presentation of fabrication of active surface layer.

This step is basically inclusion of dopamine moieties into the poly(ionic liquid)s. In air atmosphere in presence of tris buffer dopamine form cyclic radical as shown in Figure 2. This radical can self-polymerize as well as can react with the vinylic or allylic pi systems through radical coupling. This would lead to the precursor of the active layer, which can be easily attached to the poly amide support. For both the PIL polymers, a similar reaction would occur. This inclusion of dopamine moieties has been attempted based on the literature reports on dopamine-triggered polymerization of sulfobetaine methacrylate monomers [57].

#### 3.1.3. Fabrication of PIL Augmented Membrane

**Figure 3:**
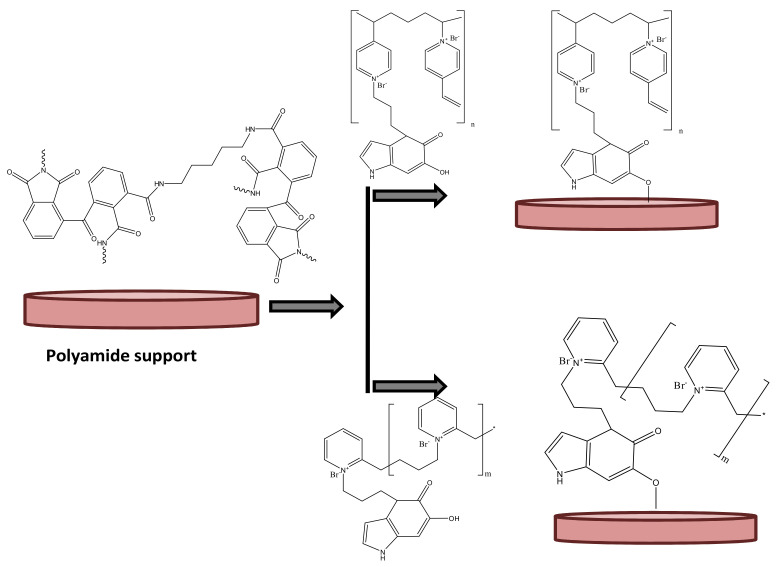
The schematic presentation of the augmentation of poly(ionic liquid) active layer on inert polyamide support.

This final fabrication of the PIL inclusion membrane basically was achieved in two steps. In the first step, the inner supportive polyamide layer was fabricated using the same protocol as reported earlier [37]. In the subsequent step, the dopamine incorporated PILs were attached on the supportive layer surface. Dopamine moieties were acting as bridging system between PIL and supportive polyamide layer. The plenty of hydroxyl groups present in dopamine moieties were believed to be involved in coupling with the amide moieties of supporting layer by forming ester bonds. Figure 3 schematically presented the coupling of PIL with polyamide supporting layer bridging through dopamine moieties. Due to the formation of covalent bond between the active PIL layers and supportive polyamide layer; the resulting membrane should acquire high degree of stability even in drastic chemical environment compared to the pressure assisted deposition of PIL layers on the same support. Since in the present case, this PIL inclusion membrane will be utilized for water-based application, so the membrane should sustain the aqueous feed solutions to be encountered.

#### 3.1.4. Measurement of Water Contact Angle

**Figure 4:**
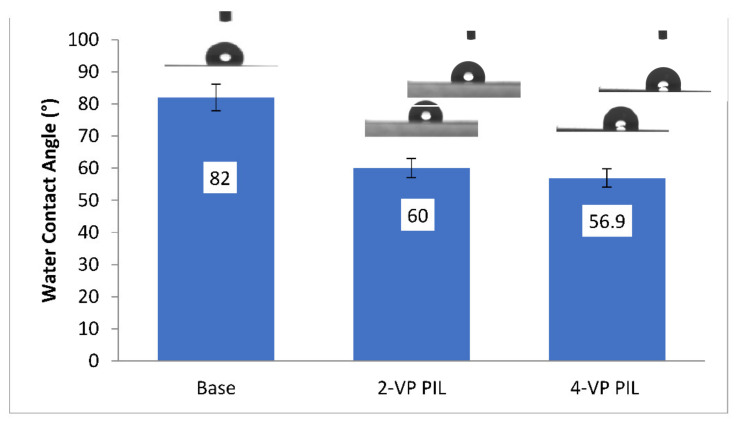
Water contact angle measurements for the Base and PIL Augmented Membranes.

The base membrane had a contact angle of 82°. The contact angle of the 2-vinyl pyridine PIL membrane and the 4-vinyl pyridine PIL membrane was found to be 60° and 56.9°, respectively.The results primarily indicated the successful modification of the base membrane. The modification was found to induce hydrophilicity on the modified membrane surface. This hydrophilic enhancement can be explained by the induction of electrostatic interaction between the water molecules and charge separation within the repetitive units of ionic liquids [34]. Figure 4 represents the water contact angles for virgin and the modified membranes.

#### 3.1.5. Zeta Potential Measurement

**Figure 5:**
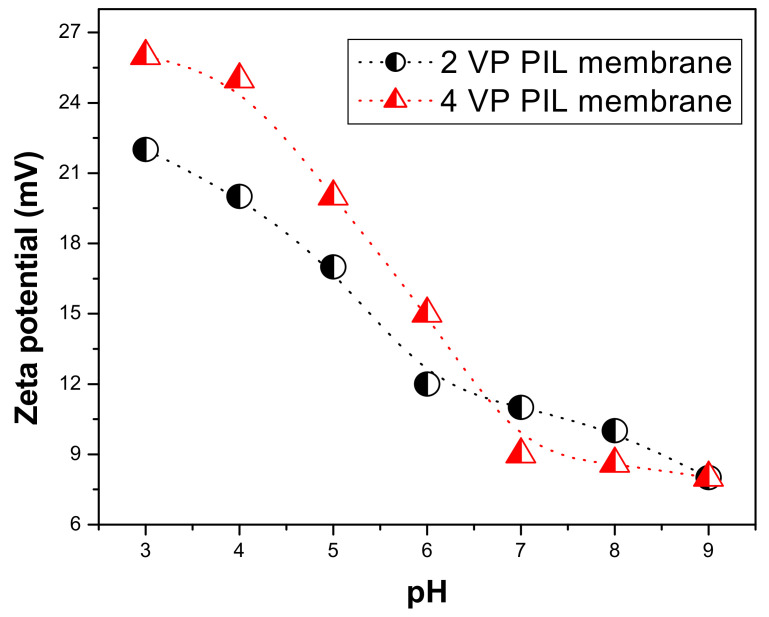
The zeta potential on PIL augmented membrane surfaces at various feed pH.

Figure 5 is showing the variation in zeta potential as a function of feed acidity on the surface of the PIL augmented membranes. At a lower pH, i.e., in acidic region the zeta potential for 4 VP PIL membrane was found to be more positive compared to that of 2 VP PIL membrane. The highly positive zeta potential can be attributed to release of mobile bromide ion from PIL moieties.Since the cationic part of the PIL moieties are attached to the membrane active layer through covalent bonds, they cannot be released from the membrane surface by breaking of bonds. However, the bromide ions, which are electrostatically attached with the PIL pyridinium backbone can be removed easily. At higher acidity, the availability of H^+^ enhanced and hence H^+^ ions compete with pyridinium ion in electrostatic interaction resulting in the release of bromide anion from membrane surface making the PIL cations in bear condition. Though below pH 7, the surface charge for 4 VP PIL membrane was found to be more than that of 2 VP PIL membrane, at pH 7, they are either same or reverse trend was observed. This high positive charge on PIL membranes also can result inter molecular H-bonding to a large extent when in contact with any protic solvent. The membrane surfaces are expected to be highly hydrophilic, which corroborates well with the water contact angles on PIL membrane surfaces.Zeta potential values at pH 7 on membrane surfaces were found to be 11.2 mV and 9.3 mV, respectively, which matched well with that of poly(ionic liquid) polymers [34]. This not only indicates the successful augmentation of poly(ionic liquid) on the membrane surface, but also revealed that the membrane surface characteristic would be very similar to that of the PIL polymers.

#### 3.1.6. FTIR Analysis for Functionality Identification on Membrane Surface

**Figure 6:**
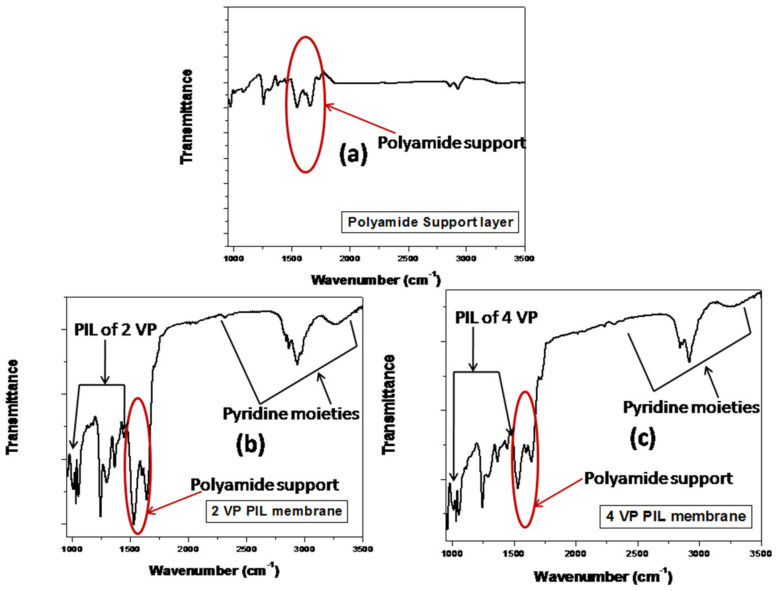
FTIR spectra for polyamide support membrane (**a**) and PIL augmented membranes (**b**,**c**).

Figure 6 shows the FTIR spectra for polyamide inert support layer and PIL augmented membranes to monitor the changes in functionalities on PIL augmentation. The polyimide base membrane and both PIL modified membranes exhibit two distinctive peaks around 1535 cm^−1^ to 1642cm^−1^ which correspond to the amide moieties from polyimide support and was seen in case of virgin membrane as well as PIL modified membranes [36]. Kamaz et al. reported the pressure-assisted deposition PILs from 2 vinyl and 4 vinyl pyridines on same polyamide support. They have reported the similar peaks 1534 cm^−1^ and 1648 cm^−1^ corresponds to polyamide moieties [37]. Yang et al. reported a series of band at 1545 cm^−1^, 1660 cm^−1^, 1723 cm^−1^ and 1778 cm^−1^, correspond to the polyamide moieties of the supportive layer [58]. The broad peak having lots of fine structure on it in the range of 2831 cm^−1^–3093 cm^−1^ was seen in case of PIL modified membranes, which was attributed to the characteristics of polyvinyl pyridine ion. The peaks in the range of 950–1050 cm^−1^ was also evidencing the attachment of polyvinyl pyridinium ion on base membrane [37]. The peaks in the range of 950–1468cm^−1^ were attributed to the PILs of 2vinylpyridine, and peaks in the range of 1214–1468cm^−1^ attributed to PIL of 4 vinyl pyridine [58,59]. Peaks in the range of 3100–3600 cm^−1^ were attributed to pyridinium ion and was also reported by Kamaz et al. and Rahim et al. [37,60].

#### 3.1.7. Microscopic Imaging for Surface Morphology

**Figure 7:**
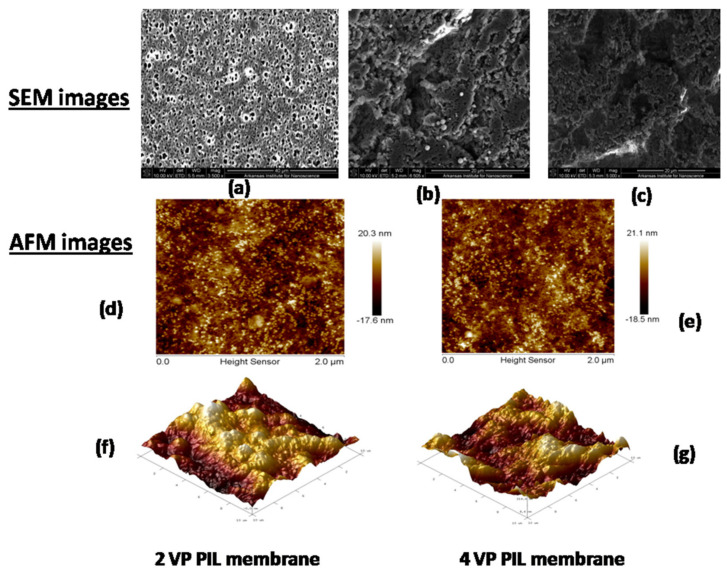
SEM Images of (**a**) Polyamide support layer; (**b**) Membranes obtained from poly(2-Vinyl pridinium bromide) augmentation; (**c**) Membranes obtained from poly(4-Vinyl pridinium bromide) augmentation; (**d**,**f**) AFM image for membrane obtained from poly(2-Vinyl pridinium bromide) augmentation; (**e**,**g**) AFM image for membrane obtained from poly(4-Vinyl pyridinium bromide) augmentation.

The appearance of dense poly(ionic liquid) active surface on inert porous polyamide support layer was evident from SEM images. The PIL inclusion membranes exhibited rough surface, which was attributed to conjugative presence of aromatic ring and pyridine moieties from poly(ionic liquid) surface layer.The mean roughness (R_a)_; root mean square of Z values (R_r_) and maximum vertical distance between the highest and lowest data points (R_max_) for PIL augmented membranes were found to be 30 nm, 45 nm and 300 nm respectively for the membrane having poly (2 vinyl pyridinium bromide) augmentation; whereas that for membrane with poly(4-vinyl pyridinium bromide) magnification were estimated as 35 nm, 50 nm and 385 nm, respectively. Similar enhancement in roughness on inclusion of poly(ionic liquid) was also reported in our earlier literature [37]. The R_a_, R_r_ and R_max_ values for the pressure assisted self-assembled 2 VP and 4 VP PIL augmented membranes were reported to 28.5 nm, 42 nm, 313 nm and 29.7 nm, 40 nm and 378 nm, respectively. Figure 7a is showing the SEM image of the supportive layer at 10 kV HV, 5.5 mm WD, 3500 magnification and 40 μm range. The image clearly indicated the highly porous nature of the supportive layer. Figure 7b,c are showing the SEM images for PIL modified membranes at 20 μm range over 5000 magnification. A dense surface was observed for both the systems, indicating change in surface morphologies. Figure 7d,e are showing the AFM images for 2 and 4 VP PIL augmented membranes as surface view, while the cross-sectional AFM images for both the modified membranes were shown in Figure 7f,g. All the images reflect the rough surface of the modified membranes. Similar observation was also reported by Kamaz et al. during pressure assisted deposition of poly(ionic liquid)s on polyamide supportive layer [37]. Figure 7f,g images were taken in 10 × 10 μm range. The grafting of PIL brushes was found to enhance the surface roughness for hollow fiber membranes [61]. The dense and rough structures revealed the successful incorporation of PIL moieties on supportive layer.

#### 3.1.8. EDX Analysis to Understand the Appearance/Relative Concentration of Heteroatom on Membrane Surface

Table 2 is showing the analytical results obtained from elemental mapping of EDX spectroscopy for the base membrane as well as PIL modified membranes.In base membrane, there was presence of C, N and O as hetero atoms in the relative composition of~77%, 15% and 7.8%, respectively. This data agreed well with the polyimide nature of the support layer. However, on modification, a drastic enhancement in N weight% was observed for both the modified membranes. Almost 1.5 to 2-time enhancement N amount can be easily correlated with incorporation of several pyridine moieties on the surface. A reduction in C and O content was also noticed in modified membranes compared to the base membrane. Additionally, ~14–15% of Br wt.% was found to appear for both the modified membranes. This was arising from the bromide ion, which was an anionic part of the poly(ionic liquid) moieties.

### 3.2. Application

#### 3.2.1. Separation of Dyes

**Figure 8:**
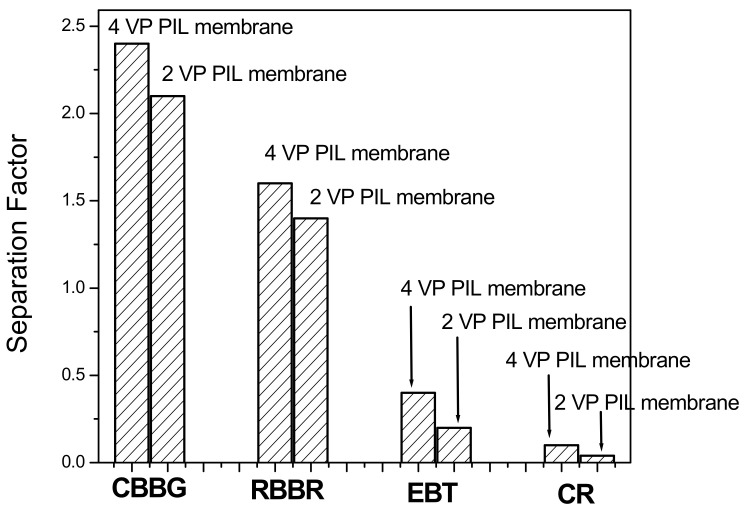
Separation factors for dyes using PIL augmented membranes.

**Figure 9:**
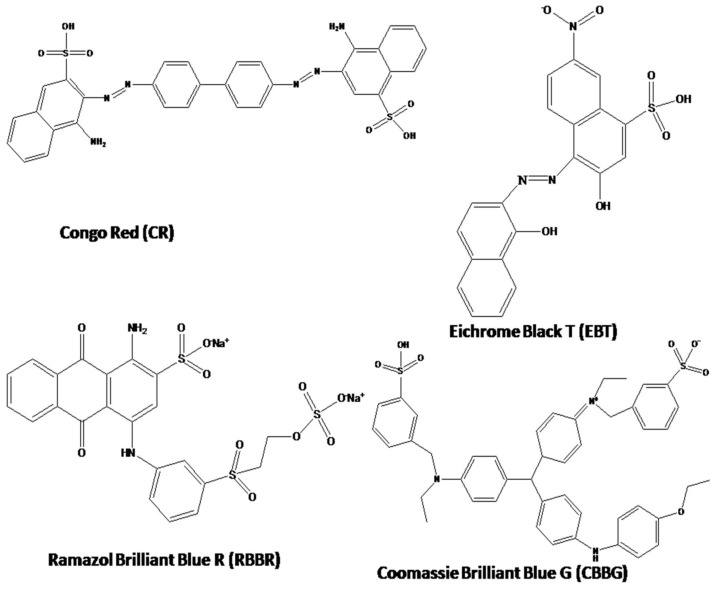
Molecular structures of the dyes utilized in present investigation.

Figure 8 is showing the separation factor of different dye solutions through PIL augmented membranes. The separation factor for i^th^ component was calculated based on the equation as follows:

(1) $${\left(S.F.\right)}_{i}=\frac{{\left[\frac{C_{i}}{C_{RBBR}+C_{EBT}+C_{CR}+C_{CBBG}}\right]}_{Permeate}}{{\left[\frac{C_{i}}{C_{RBBR}+C_{EBT}+C_{CR}+C_{CBBG}}\right]}_{feed}}$$

where, C_i_ is the component of interest, whose separation factor is evaluated. C_RBBR_, C_EBT_, C_CR_, C_CBBG_ are the concentration of those dyes. The separation factor is basically the relative concentration of the interested component in permeate to that in feed solution. From the figure it is cleared that, for all the dyes, the separation factor values in 4 VP PIL membrane are more compared to 2VP PIL membrane. This can be attributed to steric hindrance associated with vinylic group in the 2 position of the pyridinium N. In case of dyes, the molar mass for EBT was found to be 461.38 g mol^−1^, RBBR is 626.53 g mol^−1^, CR is 696.66 g mol^−1^ and CBBG is 854 g mol^−1^, respectively. If the driving force for the mutual fractionation is molar mass or molecular weight, then the trend of permeation was expected to be EBT > RBBR~CR > CBBG. However, the present trend is CBBG > RBBR > EBT > CR, indicating the predominance of the factor other than molecular weight or molar mass. The pi electron cloud-induced separation is proposed to be the driving force for fractionation of dye molecules as also reported for supported ionic liquid membrane, polyionic liquid polymeric materials and PIL membrane fabricated through pressure assisted self-assembly [21]. The ionic liquid-based membranes were reported to have high affinity towards the pi electron cloud, which was reported to be driving force for separation of hexane and benzene, fractionation of aromatics attached to either electron withdrawing or electron-donating functional groups [37]. In present case, the separation factor for the dye molecules were found to follow the order: CBBG > RBBR > EBT > CR. Figure 9 illustrates the molecular structures of these dyes. The pi electron cloud was found to be highly localized over large aromatic skeleton showing maximum separation factor in CBBG. However, in biphenyl moiety of CR, the steric repulsion between two ortho hydrogen resulted the loss of planarity between these two aromatic systems and there would be a loss of pi delocalization between these two halves of the molecule. This resulted the lowest separation factor of CR through VP PILs. In case of EBT and RBBR, the separation factor values were found to be more for the later dye, which might be attributed to the fact that, aromatic pi electron cloud in EBT was associated with electron withdrawing nitro group, while RBBR was associated with electron-donating amino group. Hence, pi electron density in EBT was lesser compared to RBBR and hence resulted such fractionation.

#### 3.2.2. Fractionation of Sugars

**Figure 10:**
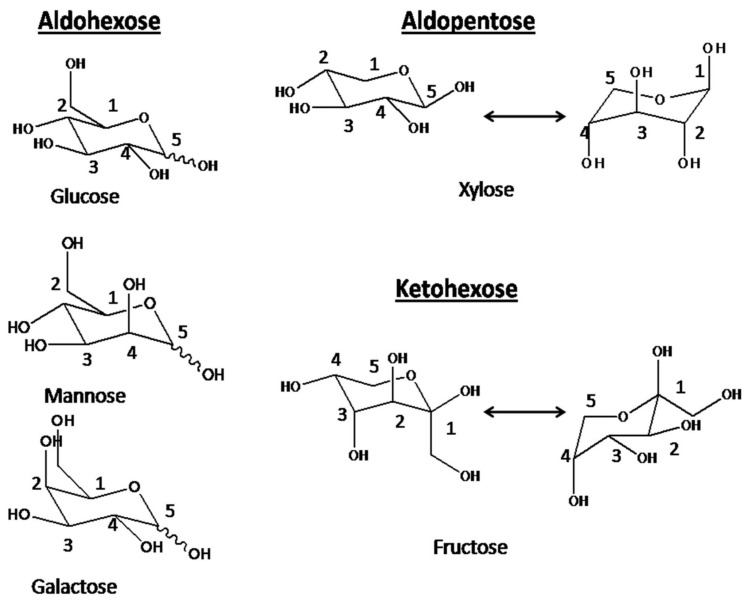
Cyclic chair structures for the sugar molecules: Glucose, Mannose, Galactose, Xylose.

**Figure 11:**
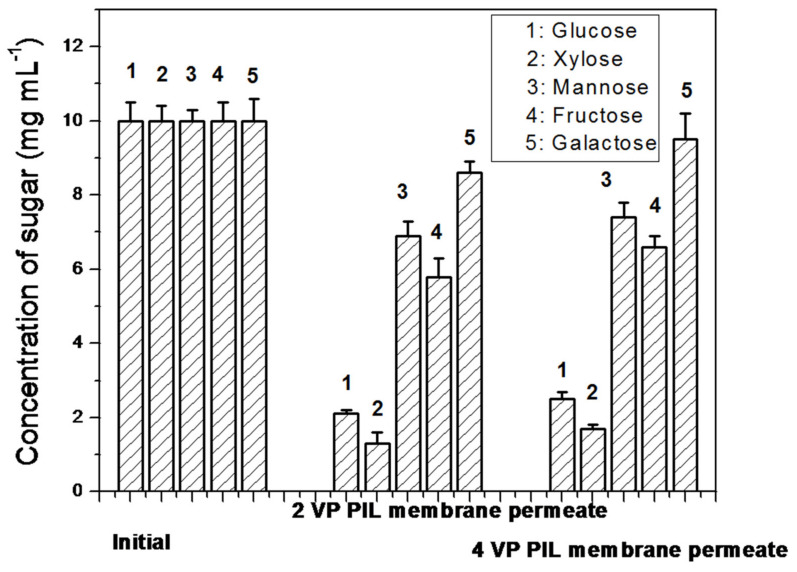
The concentrations for the sugar in initial feed and the permeate solutions obtained by filtration through PIL augmented membranes.

A mixture of 10 mg mL^−1^ of glucose, xylose, galactose, mannose and fructose were taken and was allowed to pass through the PIL membranes. The concentration of these sugar molecules in permeate were evaluated. The fractionation in sugar has been observed. The trend in permeation was found to be Galactose > Mannose > Fructose > Glucose > Xylose. Out of these sugars, Xylose is aldopentose type, i.e., five carbon sugar. therefore, based on size exclusion principle, this should have maximum permeability through any membrane as others are hexose sugar i.e., six carbon sugar. However, xylose showed the lowest permeability indicating size exclusion was not the driving force for the permeation. The molecular weight of aldohexose, i.e., glucose, galactose, and mannose and ketohexose, i.e., fructose is found to be almost same, 180.16 g mol^−1^, while the aldopentose, xylose is having 150.13 g mol^−1^. If molecular weight would play the driving role in fractionation of sugar, then xylose should show the maximum permeation, while other sugars, i.e., glucose, galactose, mannose and fructose should show same permeation and hence their mutual separation would become impossible. In reality, the trend in permeation was found to follow: Galactose > Mannose > Fructose > Glucose > Xylose, suggesting molecular weight cannot be the predominating factor for their fractionation. The chair cyclic structures (Figure 10) for these sugars have been considered for explaining the separation. For xylose totally, four hydroxyl groups can be interacted, while rest of the sugar molecules, total five hydroxyl groups can participate in interaction with pi electron cloud of poly(ionic liquid) moieties of the membranes.hence it showed the lowest permeability through PIL augmented membranes. In case of galactose, three hydroxyl groups in carbon number 1,2,3 are upward giving rise to six lone pairs of electrons nearby along with one lone pair of electrons from C-O-C cyclic O. Due to the closest proximity of these seven lone pairs of electrons, the extent of interaction with the pi electron clouds of PIL moieties are tremendous. Hence galactose showed maximum permeability.In case of mannose, three hydroxyl groups are upward but attached to the ring carbon at 1,2 and 4 positions. Hence the proximity of these lone pair electrons will be lesser compared to galactose. Hence, mannose showed 2nd highest permeability through these PIL augmented membranes. In case of fructose, which is a ketohexose, three hydroxyl groups attached to carbon 1, 3 and 4 positions can be on the same side. Hence, mannose and fructose can be expected to have similar permeation through PIL augmented membranes. However, fructose showed lower permeation compared to mannose, which might be attributed to the less reactivity of keto carbonyl compared to the aldehydic carbonyl. In glucose, hydroxyl groups either at 1,3 cyclic carbon or at 2,4 cyclic carbons are in same side, hence exhibited lower permeation compared to other aldohexose or ketohexose sugars used in the present investigation. Figure 11 is showing the concentrations of these sugars in permeate.

### 3.3. Stability of the PIL Membrane

Since, the main idea of the membrane fabrication in the present study was to attach the PIL moieties, i.e., the active layer of the membrane to the supportive polyamide layer through covalent bonding bridging by dopamine moieties. This would enhance the stability of the membrane towards aqueous application even in presence of drastic chemical environment. A high loss of ionic liquid from supported ionic liquid membranes has been evidenced through SEM and EDS analyses [62]. They have reported that more viscous the ionic liquid is more is the stability of the supported ionic liquid membrane. Fortunato et al. have reported that even after seven days immersion of supported ionic liquid membrane into aqueous feed not much ionic liquid moieties came out of PVDF supportive layer resulting in the vacant pores [63]. XPS investigation was utilized for evidencing the above fact. Dahi et al. reported a high degree of stability of supported ionic liquid membrane even after 71 h of continuous permeation at normal condition [64].

**Figure 12:**
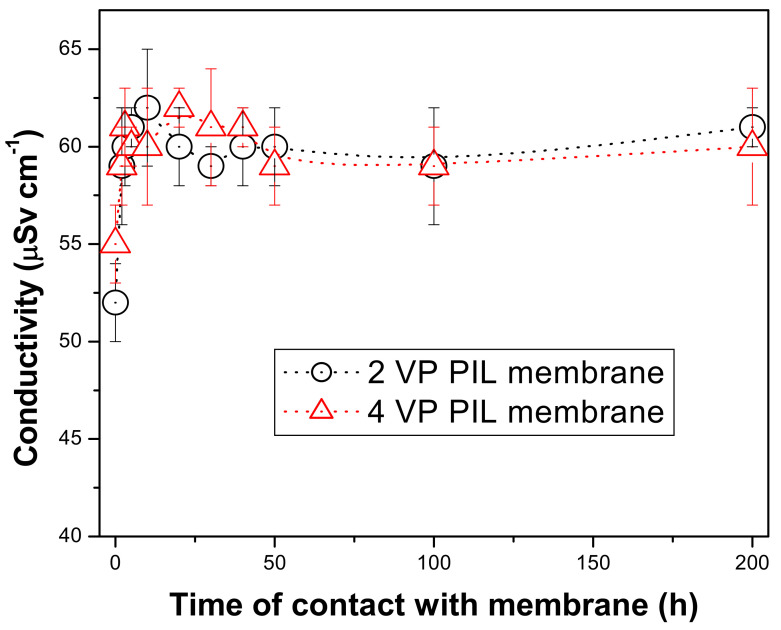
Monitoring of the conductivity of the supernatant solutions where PIL augmented membranes were immersed at various time interval.

In order to evaluate the stability of the PIL augmented membranes, the membranes were allowed to keep in contact with the water (immersed in water) for long period of time and the conductivity of the supernatant was measured with different time interval. If the PIL moieties are fragmented and come to the water, then the conductivity of the supernatant would increase as they are ionic in nature. Figure 12 is showing the experimental results. This showed that within initial 1–2 h, there were 5–10% enhancement in the conductivity of the supernatant solutions. This can be attributed to the release of some of the mobile bromide ions into the medium. This result corroborates with that obtained in zeta potential measurement of the membrane surfaces. It was also to be noticed that for both the membranes the conductivity values reached up to 60 μSv cm^−1^ and then almost no change in conductivity for up to 200 h. This revealed that no ionic fragment from PIL augmented membranes came to the medium for such long time. This revealed that these PIL augmented membranes exhibit sufficient stability towards aqueous phase application. The PIL augmented membranes formed by pressure-assisted self-assembly of 2 and 4 VP OILs showed very good stability towards non-polar and aprotic solvents. However. in aqueous medium or highly polar solvents, some of the PIL fragments were found to get dissolve into the aqueous feed solution as a result, enhancement in conductivity was noticed for such cases [37]. However, the covalent bonds between the supportive polyamide layer and PIL active layers through polydopamide bridge removed such aqueous phase instability of the membranes. Hence in the present case, PIL augmented membranes were found to be suitable for long-term application.

## 4. Conclusions

Novel poly(ionic liquid) based membranes were fabricated on polyamide support layer by bridging through dopamine. The PIL augmentation was found to enhance the hydrophilicity of the membrane surface. In presence of aqueous environment, the mobile bromide ions were found to get released into the medium, resulting in highly positive surface charge as revealed by zeta potential measurement. The appearance of pyridinium peaks in FTIR revealed the attachment of pyridinium based PIL on membrane surface. The appearance of bromide atom and enhancement of nitrogen relative concentration in EDX spectra also indicated the successful augmentation. These membranes were utilized for the fractionation of dye molecules based on the pi-pi interaction. Dye molecules with more extended delocalization of aromaticity or more pi electron density was found to interact strongly with the aromatic pi electron clouds from PILs and hence was permeated to a greater extent. The trend in dye permeation followed the order: CBBG> RBBR> EBT> CR. A clear fractionation in sugar molecules were also demonstrated based on pi electron interaction. The aldohexoses with six hydroxyl groups were found to be permeated higher extent compared to aldopentose with five hydroxyl groups, even though the former is six carbon molecules, while later is five carbon molecules.More hydroxyl groups in same side of the chair structure of sugar molecules were found to interact strongly with PIL moieties resulting in more permeability of those sugars. Between similar configuration, aldohexose showed stronger interaction and hence better permeability compared to ketohexose type of sugars.

## Figures and Tables

**Table 1:** Summary of the physiochemical properties of the dyes studied.

Properties	EBT	RBBR	CR	CBBG
Chemical Formula	C_20_H_12_N_2_NaO_7_S	C_22_H_16_N_2_Na_2_O_11_S_3_	C_32_H_22_N_6_Na_2_O_6_S_2_	C_47_H_48_N_3_NaO_7_S_2_
Molecular Weight	461.38 g/mol	626.53 g/mol	696.66 g/mol	854.05 g/mol
Solubility Water (g/L)	50	980	~30	50
Maximum Wavelength Absorbance (nm)	503	592	490	467
Topological Polar SA (Å^2^)	173	255	233	153
Color	Black	Dark Blue	Red-Brown	Dark Red-Blue

**Table 2:** EDX data for polyimide support membrane and PIL augmented membranes.

Membrane	Element	Wt.%	Error
Support layer	C	77.07	11.1
	O	7.83	3.5
	N	15.10	3.7
2 VP PIL membrane	C	49.16	13.2
	O	3.79	1.7
	N	33.23	3.0
	Br	13.82	3.96
4 VP PIL membrane	C	55.07	13.2
	O	4.46	1.2
	N	25.23	3.1
	Br	15.00	4.2

## Data Availability

Not applicable.
